# The chemical ladies

**DOI:** 10.1038/s42004-020-0296-z

**Published:** 2020-05-01

**Authors:** Victoria Richards

**Affiliations:** Communications Chemistry, https://nature.com/commschem

**Keywords:** Chemistry, Materials science, Energy science and technology, Nanoscience and technology

## Abstract

In celebration of International Women’s Day, on 6 March 2020 the University of Nottingham hosted its second Women in Chemistry conference. Or, as branded by a security guard at the host building, a ‘chemical ladies’ meeting’. Victoria Richards recounts the event and shares key take-home messages from the speakers.

The University of Nottingham’s second Women in Chemistry conference^1^ was well-attended by a mix of academic and industrial chemists, and the atmosphere was like no other. It is incredibly empowering to walk into a room full of scientists and to be a member of the majority, and the enthusiasm and engagement from the delegates would suggest that this sentiment was widely shared. Opportunities for discussion, networking, scientific curiosity and laughter were rife, and the aim of celebrating the successes of women in chemistry and creating discourse around gender equality in STEM resonated throughout the day.

Kicking off the talks, Dr. Jenny Zhang (University of Cambridge) described how switching continents, cultures and language at a young age prepared her for versatility later in life and taught her to always question societal norms. Coming to the end of her Ph.D. in medicinal chemistry, she decided that she wanted to change directions and move into energy research. She was advised against doing so by many, who warned of a competitive landscape, or commented on the risk of leaving a field in which she was already succeeding, but decided to pursue her aspirations nonetheless. Jenny reflected that changing fields was likely the biggest challenge that she has faced in her career to date—coming up against grant interview panels of solely white men also featured—but found that taking calculated risks, alongside a combination of patience, hard work, creativity and collaboration, paid dividends for her. Indeed, it was hard not to be impressed by her contributions to the field of semi-artificial photosynthesis^2^. Words of advice to her captivated audience were that they should not be afraid of being different (and that they should instead turn what makes them different into a superpower), and to not be afraid to try new things and evolve.

Professor Serena Corr (University of Sheffield), who we learnt released a music album with an Irish band during her postdoc in California, spoke to the fact that the most successful teams are those that bring diverse people from varied backgrounds together. One of her academic interests is studying foreign materials present in the wood of the Mary Rose^3^, a historic Tudor Warship that was lost to sea for 400+ years, and Serena walked us through how a team of scientists, led by Professor Eleanor Schofield at the Mary Rose Trust, from diverse academic backgrounds—synthetic chemical nanoscience, polymer chemistry, and materials science, among others—are innovatively collaborating on the conservation of this antique.

The development of next generation cathode materials for Li-ion batteries is another focus of Serena’s, and here too she spoke of the importance of a diverse team, naming a Faraday Institution consortium that brings together electrochemists, synthetic chemists, computational chemists and materials scientists from both academia and industry, with diversity of background, experience and gender.

Serena spoke of her experience with the two body issue—where two academics in a relationship need to be employed at the same or neighbouring institutions if they are to share a life in the same location—and encouraged raising it early on in job applications. She suggested rebranding this from the better-known two body ‘problem’ to a more addressable ‘issue’. She also opened up about misinformed perceptions about pregnancy and advocated for calling out unacceptable behaviours and practices. She demonstrated that a research career can continue to thrive during pregnancy, citing experiments performed with colleagues at an X-ray synchrotron, and stressed the importance of nurturing a supportive environment. More broadly, she talked about the slow road towards female to male parity in academic research, and gave sage advice on what fellow chemists, of all genders, can do to accelerate reaching this: demanding more from senior academics who are in a position to influence policy, encouraging women and other minorities to put themselves forward, and helping to give greater visibility to role models who are actively working for change, all featured highly.

Dr. Samantha Hughes (AstraZeneca, Cambridge) titled her talk ‘navigating the career maze’, and it was perfectly clear why: in the 18 years since obtaining her Ph.D., she has faced many forks in the road—owing to the two body issue; at the point of deciding between academia and industry; and once as a result of redundancy. In all cases there was no obvious path to follow. Rather, navigating career options required a careful weighing of professional, personal and circumstantial factors, with her passion for computational medicinal chemistry driving each of her decisions^4^. Samantha noted that her transition to industry came further down the line than it could have, likely owing to a lack of concrete insight into the pharmaceutical industry. Indeed, in academia, a career as a PI can often be the only real picture of success that one is accustomed to, and without access to role models in alternative careers, the path less trodden can be a harder one to find. She advised that each individual should carefully reflect on their own scientific and career drivers, should remember that calculated risks can pay off—and can often provide the biggest opportunities for development—and that one should always be prepared to adapt to change.

Professor Elena Besley (University of Nottingham) spoke of her time at Saint Petersburg State University when she was one of just a few women in a large group of physics undergraduate students, all of whom had never been taught by a female lecturer. She reflected on the progress towards equality that has been achieved since then, and used much of her talk to highlight six excellent female students who spent the fourth year of their MSci undergraduate studies working in her research group. We learnt of their scientific successes at Nottingham^5–10^, their career paths thereafter, and key lessons that they took with them from their time in the group. The important skills that they acquired from coding, scripting, data analysis and developing new software are now put to good use in their current roles: spanning forensic services, postgraduate research, chemical industry and management. Take-away career advice from Elena was to follow your passion, be flexible, and be prepared to drop jobs that you do not feel attached to.

After describing some of her team’s recent adventures with halogenases^11^, Professor Rebecca Goss (University of St Andrews), our final speaker of the day, beautifully narrated her academic highs and lows. She spoke of the privilege of having a career where you can mentor younger team members and pursue dreams and ideas. She contrasted becoming a lecturer at age 26 with the imposter syndrome that followed, presented a stark picture from a Royal Society of Chemistry awards dinner in 2007 in which she was the only woman of 31 awardees (noting that the RSC have subsequently worked tirelessly to address this), and stated that she was the first woman to be appointed Professor of Organic Chemistry at St Andrews. Rebecca encouraged junior members to embark on what she deemed to be an exciting and rewarding career, and suggested that new academics balance low-risk projects alongside more ambitious ones, ask peers to pre-review grant applications, and perhaps most importantly, choose an advisor wisely, because ‘when you choose your advisor, you choose your advice’.

In my own talk I described the role of a scientific editor at Nature Research, and outlined some of the responsibilities that editors have towards women in science. A lack of bias in decision making is obvious, and proactive thinking about gender balance when it comes to commissioning content, inviting peer reviewers, selecting speakers for conferences, inviting researchers to join editorial boards, awarding travel grants or poster prizes, and nominating reviewers for additional recognition, are some of the ways in which we can ensure that women are invited to the same playing field as men. Like Samantha I spoke of my transition from academia to industry, though mine was much more abrupt, with my postdoc experience lasting only 1 week as opposed to 18 months. A difficult time to reflect back on, but plenty of lessons learnt, including listen to your gut; don’t blindly continue down a path that you know you have little long-term interest in; and just because you find that you can do something that resembles success, it doesn’t necessarily mean that you should.

After an intense but inspiring day, a panel discussion, chaired by Professor Steve Howdle (Head of The School of Chemistry at the University of Nottingham), covered a series of topics chosen by the audience. Work/life balance, imposter syndrome, calling out unacceptable behaviours, feeling isolated in a male-dominated environment, and the importance of male involvement in discussions on gender equality were the focus of the conversation. While I cannot say that we solved all or even any of these issues, the discourse was refreshingly honest, and there was plenty of food for thought to take away from the day.

Congratulations to the organizers—Marysia Tarnowska, Ellen Guest, Charlotte Roy, Megan Thomsett, Ana Alves Costa Pacheco, Bruna Lacerda Da Silva Abreu and Patrick Morgan—for putting on such a fantastic event. I was personally blown away by the spirit of the ~200-strong delegation and their collective sense of urgency on the road to gender equality in the chemical sciences. I look forward to seeing the progress made, and the achievements of many of these talented chemists, in the years to come.

## References

[1] Kundu S (2019). Women in chemistry. Nat. Chem..

[2] Zhang JZ, Reisner E (2020). Advancing photosystem II photoelectrochemistry for semi-artificial photosynthesis. Nat. Rev. Chem..

[3] Peplow M (2018). A conversation with Serena Corr. ACS Cent. Sci..

[4] Hughes SJ (2011). Fragment based discovery of a novel and selective PI3 kinase inhibitor. Bioorg. Med. Chem. Lett..

[5] Algara-Siller G (2013). Electron-beam engineering of single-walled carbon nanotubes from bilayer graphene. Carbon.

[6] Henley A (2016). Computational evaluation of the impact of incorporated nitrogen and oxygen heteroatoms on the affinity of polyaromatic ligands for carbon dioxide and methane in metal–organic frameworks. J. Phys. Chem. C.

[7] Lennox MJ (2017). The right isotherms for the right reasons? Validation of generic force fields for prediction of methane adsorption in metal-organic frameworks. Mol. Simul..

[8] Bolotov VA (2018). Enhancement of CO_2_ uptake and selectivity in a metal-organic-framework by the incorporation of thiophene functionality. Inorg. Chem..

[9] Henley A (2016). Effective binding of methane using a weak hydrogen bond. J. Phys. Chem. A.

[10] Skowron ST (2019). The effects of encapsulation on damage to molecules by electron radiation. Micron.

[11] Gkotsi DS (2019). A marine viral halogenase that iodinates diverse substrates. Nat. Chem..

